# FAPI PET/CT Imaging—An Updated Review

**DOI:** 10.3390/diagnostics13122018

**Published:** 2023-06-09

**Authors:** Kunal Ramesh Chandekar, Arun Prashanth, Sobhan Vinjamuri, Rakesh Kumar

**Affiliations:** 1 Department of Nuclear Medicine, All India Institute of Medical Sciences, New Delhi 110029, India; kunal.chandekar@aiims.edu; 2 Department of Nuclear Medicine, MIOT International Hospital, Chennai 600089, India; dr.arunprashanth@gmail.com; 3 Department of Nuclear Medicine, Royal Liverpool and Broadgreen University Hospital, Liverpool L7-8YE, UK; sobhan.vinjamuri@gmail.com

**Keywords:** fibroblast activation protein (FAP), FAPI, PET/CT, imaging, diagnostic, theranostics

## Abstract

Despite revolutionizing the field of oncological imaging, Positron Emission Tomography (PET) with [^18^F]Fluorodeoxyglucose (FDG) as its workhorse is limited by a lack of specificity and low sensitivity in certain tumor subtypes. Fibroblast activation protein (FAP), a type II transmembrane glycoprotein, is expressed by cancer-associated fibroblasts (CAFs) that form a major component of the tumor stroma. FAP holds the promise to be a pan-cancer target, owing to its selective over-expression in a vast majority of neoplasms, particularly epithelial cancers. Several radiolabeled FAP inhibitors (FAPI) have been developed for molecular imaging and potential theranostic applications. Preliminary data on FAPI PET/CT remains encouraging, with extensive multi-disciplinary clinical research currently underway. This review summarizes the existing literature on FAPI PET/CT imaging with an emphasis on diagnostic applications, comparison with FDG, pitfalls, and future directions.

## 1. Introduction

The field of oncology has been revolutionized by molecular imaging which assesses tumor biology as opposed to conventional radiologic imaging which focuses on morphological anatomy [1]. Molecular imaging permits non-invasive visualization of physiological or pathological processes at the cellular or sub-cellular level. Positron emission tomography/computed tomography (PET/CT) is a hybrid imaging tool that provides complementary functional and structural information [2]. [^18^F]Fluorodeoxyglucose (FDG), first developed in the late 1970s as a tracer to map regional cerebral metabolism, is now the most universally used PET tracer having a myriad of oncological and non-oncological applications [3,4]. Despite its undeniable clinical utility, FDG uptake is a surrogate for glucose transport/metabolism and is not specific for malignancy. Subsequent translational research led to the development of more specific tracers such as radiolabeled somatostatin receptor (SSTR) targeting agents and prostate-specific membrane antigen (PSMA) ligands, which have successfully been incorporated into modern-day management practices of neuroendocrine tumors and prostate cancer, respectively [5]. The ever-evolving search for cellular targets led to the discovery of fibroblast activation protein (FAP), a transmembrane glycoprotein expressed on activated fibroblasts such as cancer-associated fibroblasts (CAFs) [6]. Preliminary evidence has generated raging scientific interest in FAP as the next billion-dollar pan-cancer target in the field of nuclear medicine [7]. Several radiolabeled fibroblast activation protein inhibitor (FAPI) tracers are currently being investigated as PET imaging agents for various neoplasms with the potential for theranostic application. This review summarizes the existing literature on FAPI PET/CT imaging with an emphasis on diagnostic applications, comparison with FDG, pitfalls, and future directions.

## 2. Understanding Tumor Biology

In addition to neoplastic cells, tumors contain stromal components which form the tumor microenvironment (TME) [8]. The TME is a vastly heterogeneous system comprising CAFs, tumor-associated macrophages, immune cells, endothelial cells, and extracellular matrix (ECM). CAFs are often the most abundant cell type in the TME. CAFs are activated fibroblasts, originating from various benign cells such as fibrocytes, endothelial cells, adipocytes, and others. They have augmented proliferative and migratory capacity. They release growth factors and proinflammatory cytokines, such as transforming growth factor-β (TGF-β), vascular endothelial growth factor (VEGF), and interleukin-6 (IL-6). This complex biochemical crosstalk between the non-neoplastic cells and neoplastic cells drives metabolic reprogramming of the tumor and regulates its growth, invasion, angiogenesis, immunosuppression, and drug resistance [9]. CAFs are different from quiescent fibroblasts, being stellate-shaped and biologically active with selective over-expression of surface markers such as α-smooth muscle actin, platelet-derived growth factor-β, and FAP, which seems to be the most specifically upregulated [10].

FAP is a type II transmembrane glycoprotein consisting of 760 amino acids. FAP is a serine protease, and unlike other members of the dipeptidyl peptidase (DPP) family, it has both endopeptidase and exopeptidase activity, which enable it to cleave gelatin and type I collagen and play an important role in ECM remodeling. DPPIV shares about 50% similarity in amino acid sequence with FAP and 70% homology of the catalytic domain [11]. CAFs with FAP expression are found in various neoplasms, particularly epithelial cancers, and malignancies with a strong desmoplastic reaction such as breast, colorectal, pancreatic, and lung cancer. FAP expression has also been reported on the neoplastic cells in certain tumors [12,13]. Overall, a high degree of FAP expression is associated with tumor aggressiveness and poor prognosis [14,15]. The negligible expression of FAP in normal healthy adult tissues makes it an attractive target for oncological imaging and therapy [16].

## 3. Development of FAP-Targeting Radiopharmaceuticals

FAP overexpression has been targeted in experimental cancer imaging and therapeutics using antibodies, peptides, enzymatic inhibitors, vaccines, immuno-conjugates, and chimeric antigen receptor T cells [17]. The first clinical experience of FAP-targeted scintigraphic imaging was reported by Welt et al. in 1994, using the ^131^I-labeled murine monoclonal antibody (mAb) F19 in patients with metastatic colorectal cancer (CRC) [18]. Subsequently, sibrotuzumab, a humanized version of mAb F19, was developed to explore the possibility of FAP-targeted therapy. However, a phase I study reported that sibrotuzumab did not lead to any objective tumor response in 26 patients with advanced FAP-expressing CRC or lung cancer, hindering further development of these molecules [19]. Radiolabeled antibodies are also limited by a high molecular mass that leads to slow tracer clearance in- vivo, resulting in higher background signal, lower sensitivity for focal detection, and higher overall radiation exposure [20]. Some of the early work on FAPIs focussed on pyrrolidine-2-boronic acid derivatives such as talabostat mesylate (PT-100), an oral amino boronic dipeptide. Talabostat exhibited an affinity for members of the DPP subfamily but was not very specific for FAP [21]. Ultimately, this shifted focus to small-molecule FAP inhibitors with an N-(4-quinolinoyl)-Gly-(2-cyanopyrrolidine) scaffold, first developed at the University of Antwerp, as they had favorable pharmacokinetics and higher FAP-specificity [22,23]. These were further modified by the Heidelberg group in Germany to develop FAPI-01 and FAPI-02 as the first quinoline-based FAPIs, which were radiolabeled with ^125^I and ^68^Ga/^177^Lu, respectively. ^125^I-labeled FAPI-01 exhibited a lower degree of binding and uptake in human FAP-expressing cells in- vitro and in- vivo compared to [^68^Ga]Ga-FAPI-02, owing to its time-dependent efflux and enzymatic deiodination [24]. Lindner et al. synthesized multiple FAPI derivatives (FAPI-03 to FAPI-15) based on a common pharmacophore (UAMC1110). Of these, FAPI-04 was found to be the most promising tracer for theranostic application, having higher effective tumor accumulation (3.0% vs. 1.12% ID/g, respectively, at 24 hours) and longer retention time than FAPI-02 [25]. Further attempts at improving tumor retention led to the development of FAPI-46 [26]. At present, most of the published research studies regarding the clinical use of FAP targeting have employed FAPI-04 and FAPI-46.

Moon et al. developed novel FAPI radiotracers using squaric acid (SA)-based linker moieties between bifunctional chelators (DOTA, DATA^5m^, DOTAGA) and the UAMC1110 motif, to simplify the complex synthesis of previous chelator-based FAPIs [27]. Monomeric DOTA.SA.FAPI labeled with ^68^Ga exhibited the most favorable properties for imaging. However, its therapeutic counterpart labeled with the beta-emitting radionuclide ^177^Lu showed significant tracer washout by 48 h post-injection, limiting its clinical utility. Subsequently, the same group developed dimeric systems such as DOTA.(SA.FAPI)_2_ and DOTAGA.(SA.FAPI)_2_ to improve tumor affinity and retention times [28]. When labeled with the ^177^Lu, these dimers showed promising results for therapeutic application in an early clinical study [29]. Xu et al. developed TEFAPI-06 and TEFAPI-07 by conjugating two albumin binders, 4-(*p*-iodophenyl) butyric acid moiety and truncated Evans blue moiety, respectively, to the parent molecule, FAPI-04 [30]. An American group developed a trifunctional inhibitor, RPS-309, which has a FAP-targeting moiety, an albumin-binding group, and DOTA for radiometal chelation. Onco-FAP, an ultra-high affinity small organic FAP ligand, has been successfully labeled with ^68^Ga and ^177^Lu using DOTAGA as the chelator. The development of these molecules helped overcome rapid bloodstream clearance and improved tumor retention times in- vitro and in- vivo [31,32].

Radiolabeling with ^18^F offers several advantages over ^68^Ga such as larger batch size, lower positron energy leading to better spatial resolution, and longer half-life enabling transport to satellite centers. Toms et al. used a copper-catalyzed cycloaddition to obtain the glycosylated FAPI tracer, [^18^F]FGlc-FAPI [33]. The higher plasma protein binding and higher lipophilicity of [^18^F]FGlc-FAPI leads to slower clearance from the blood pool compared to [^68^Ga]Ga-FAPI-04. The Heidelberg group developed FAPI-74 by replacing the chelator DOTA in FAPI-02 with NOTA, which enables radiolabeling with both ^68^Ga and ^18^F. Additionally, NOTA can also be labeled with ^18^F via aluminum fluoride (AlF) chemistry, as seen in [^18^F]AlF-FAPI-74. However, [^18^F]AlF-FAPI-74 was reported to have lower specificity activity when compared to its ^68^Ga-labeled counterpart, which could potentially affect image quality [34]. Novel tracers have also been developed for single photon emission computed tomography (SPECT) imaging such as ^99m^Tc-labeled FAPI-34, ^99m^Tc-labeled FAP-targeting ligand (FL-L3), and ^111^In-labeled QCP02. In addition to ^177^Lu, FAPI molecules have also been radiolabeled with ^188^Re, ^90^Y, and ^225^Ac for therapeutic application [35,36,37,38,39].

Recently, a German group developed FAP-2286 which is a novel FAP-binding peptide, unlike the above-discussed small-molecule inhibitors of FAP. Radiotracers based on FAP-2286 have been evaluated at preclinical and clinical levels demonstrating selectivity towards FAP, high tumor uptake, high tumor retention, potential anti-tumor activity, and acceptable toxicity [40,41,42].

## 4. FAPI PET/CT Imaging—Overview

**Figure 1:**
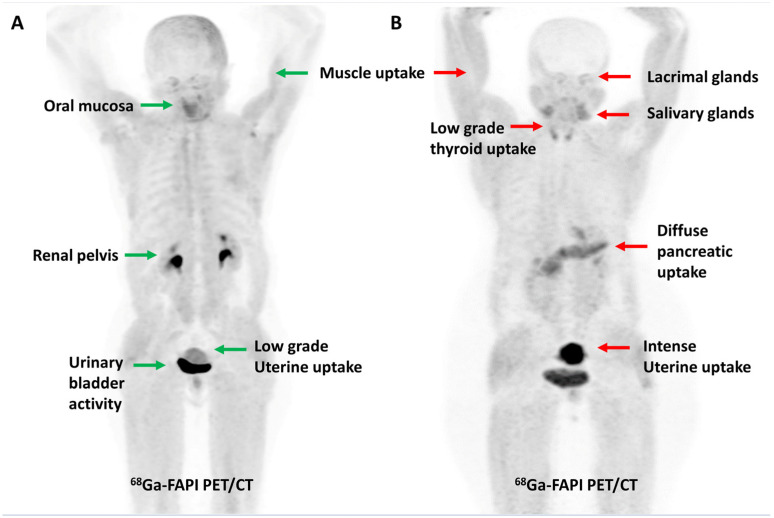
Maximum intensity projection PET images demonstrating the physiological distribution of [^68^Ga]Ga-FAPI-04 in two representative cases. Low-grade uterine uptake (green arrow) noted in a 55-year-old post-menopausal woman (**A**) is in stark contrast to the intense uterine uptake (red arrow) noted in a 42-year-old premenopausal woman (**B**).

FAP expression has been reported in over 90% of epithelial neoplasms [43]. As tumor lesions grow, they need a supporting stroma for their sustenance. Given that stroma volume can be larger than the volume of neoplastic cells, FAP-targeted PET imaging may be more sensitive than FDG PET imaging for detecting small lesions or for lesions with negligible or heterogeneous glucose metabolism. From a feasibility point of view, the ability to acquire scans as early as 10 min post-injection, lack of fasting requirements, or the need to withhold insulin/steroids makes FAPI PET imaging easier to perform, particularly in the diabetic and pediatric populations. The physiological biodistribution of FAPI tracers typically includes the uterus, spleen, lungs, heart, pancreas, oral mucosa, salivary glands, thyroid, and liver, with predominant urinary excretion, as shown in Figure 1 [44]. Radiation dosimetry studies have found that ^68^Ga-labeled FAPI tracers (FAPI-02, FAPI-04 and FAPI-46) typically result in a whole-body effective dose ranging from 1.5 to 4mSv (per ~200 MBq of tracer), which is comparable to or lower than the commonly used tracers such as FDG, [^68^Ga]Ga-PSMA-11, and [^68^Ga]Ga-DOTATATE. A lower average effective whole-body dose has been reported with [^68^Ga]Ga-FAPI-46 than with [^68^Ga]Ga-FAPI-04 (7.80 × 10^−3^ vs. 1.27 − 1.64 × 10^−2^ mSv/MBq, respectively). The urinary bladder wall has consistently been reported as the organ with the highest absorbed dose for various ^68^Ga-labeled FAPI tracers. For [^68^Ga]Ga-FAPI-04, Wang et al. reported that the organ with the highest mean absorbed dose was the urinary bladder (1.45 × 10^−1^ mGy/MBq), followed by uterus, kidneys, lungs, spleen, heart wall, and pancreas, in descending order [45,46,47,48,49,50].

## 5. FAPI PET/CT—Oncological Indications

**Figure 2:**
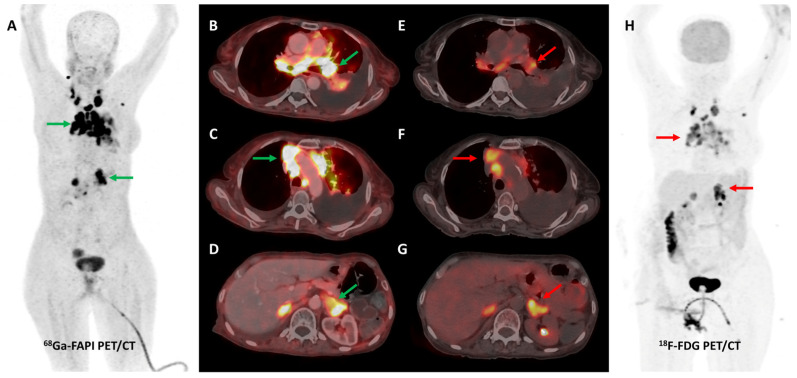
[^68^Ga]Ga-FAPI-04 and FDG PET/CT images in a 59-year-old woman with biopsy-proven metastatic left lung adenocarcinoma. [^68^Ga]Ga-FAPI-04 PET/CT images revealed intensely tracer avid left hilar mass lesion ((**B**)—green arrow), multiple enlarged mediastinal ((**A**,**C**)—green arrows) lymph nodes, and bilateral adrenal metastases ((**A**,**D**)—green arrows depicting left adrenal lesion). Additionally, moderate left-sided pleural effusion with associated left lung lower lobe collapse was noted. Overall, FAPI PET/CT demonstrated higher tracer avidity and TBRs than FDG PET/CT ((**E**–**H**)—red arrows).

Loktev et al. conducted a proof-of-concept study in 2018 where they first demonstrated high lesional tracer uptake in three patients with breast, lung, and pancreatic cancers on FAPI PET imaging [24]. Subsequently, the same group from Heidelberg reported [^68^Ga]Ga-FAPI-04 PET/CT results of 80 patients with 28 different tumor types. The degree of tracer uptake differed significantly among tumor types, being highest in sarcoma, cholangiocarcinoma, esophageal, breast, and lung cancer [51]. Few other basket trials evaluated FAPI PET/CT in heterogeneous, oncological patient cohorts. The most consistent advantage of FAPI over FDG across these studies was easier lesion detection owing to a significantly lower background signal, with resultant higher target-to-background ratios (TBRs) and sharp image contrast [52]. One such example is presented in Figure 2.

### 5.1. Hepatobiliary Tumors

Hepatocellular carcinoma (HCC) and cholangiocarcinoma (CCA) are the most frequent types of primary liver cancer [53]. Imaging-based radiologic criteria are accepted for diagnosis of HCC without confirmatory pathology [54]. However, molecular imaging with FDG PET/CT has poor sensitivity for the detection of primary liver cancer, particularly in cases of moderate to well-differentiated HCC, which exhibit high FDG-6-phosphatase activity, high expression of P-glycoprotein, and low expression of glucose transporters (GLUT1/2) [55]. To overcome these limitations, tracers such as [^11^C]acetate, [^11^C]choline, and [^68^Ga]Ga-PSMA-11 were evaluated in the detection of HCC [56,57,58]. Owing to the abundance of CAFs in the tumor stroma, FAPI PET/CT has recently been tried in HCC and CCA.

In a pilot study by Shi et al., [^68^Ga]Ga-FAPI-04 PET/CT was performed in 25 patients with hepatic nodules suspicious of malignancy. Intra-hepatic malignant lesions showed a high degree of tracer uptake; the mean SUVmax was 8.36 ± 4.21, and the mean TBR was 13.15 ± 9.48. On immunohistochemistry (IHC), prominent FAP expression was noted in 75% of the primary intrahepatic HCC lesions, being higher in poorly differentiated forms [59]. A subsequent prospective study reported that [^68^Ga]Ga-FAPI-04 PET/CT had comparable specificity and higher sensitivity (100% vs. 58.8%) than FDG PET/CT in 17 patients with primary hepatic tumors. Lesion SUVmax and TBRs were significantly higher on FAPI than FDG PET/CT [60]. These findings were confirmed by a different group in a larger study with 34 patients (20—HCC, 12—intra-hepatic CCA, 2—benign). For the detection of primary hepatic tumors, [^68^Ga]Ga-FAPI-04 PET/CT (96%) demonstrated similar sensitivity as contrast-enhanced-CT (CECT) (96%) and liver magnetic resonance imaging (MRI) (100%) but clearly outperformed FDG PET/CT (65%). Overall tumor detection rate of FAPI PET/CT was significantly higher than that of FDG PET/CT (87.4% vs. 65.0%, *p* < 0.001). [61]. Zhang et al. found that ^18^F-labeled FAPI PET/CT could differentiate HCC from benign non-inflammatory focal liver lesions (FLLs) but not from benign inflammatory FLLs owing to considerable overlap in lesional tracer uptake [62]. However, a proof-of-concept study found that kinetic parameters obtained by dynamic [^68^Ga]Ga-FAPI-04 PET/CT imaging can help in the non-invasive differentiation of HCC lesions from non-HCC lesions and healthy liver parenchyma [63].

### 5.2. Pancreatic Cancer

Pancreatic ductal adenocarcinoma (PDAC) is an aggressive tumor with a dismal prognosis [64]. Shi et al. reported that ~70–75% of PDAC specimens exhibit strong FAP expression, both on tumor cells and CAFs [65]. FAP expression is associated with desmoplasia, metastatic spread, and worse clinical outcomes in PDAC [66]. Deng et al. reported a case of pancreatic carcinoma where [^68^Ga]Ga-FAPI PET/CT led to improved detection of liver and bone metastases compared to FDG [67]. Shou et al. highlighted the additive utility of [^68^Ga]Ga-FAPI-04 PET/MR in differentiating autoimmune pancreatitis from pancreatic malignancy compared to FDG PET/CT [68]. Röhrich et al. conducted the first pilot study to assess the clinical impact of [^68^Ga]Ga-FAPI (FAPI-04 or FAPI-46) PET/CT in 19 patients with PDAC (7—initial staging and 12—restaging). FAPI PET/CT led to changes in the tumor-node-metastases (TNM) stage in 52.6% (10/19) of patients. FAPI PET/CT led to changes in management in 36.8% (7/19) of patients. The authors also reported that imaging at delayed time points could help in differentiating PDAC from pancreatitis owing to differential tracer kinetics [69]. Liermann et al. reported distinct differences in the gross tumor volume (GTV) manually contoured by 6 different radiation oncologists, based on CECT images, in 7 cases of locally recurrent pancreatic cancer having mean dice similarity coefficients between 0.55 and 0.65. GTV sizes contoured by radiation oncologists differed by more than 100% in some cases. Due to its high tumor-to-background contrast, [^68^Ga]Ga-FAPI-04 PET/CT-based automated target volume delineation (TVD) could minimize this inter-individual variability [70].

### 5.3. Colorectal and Other Gastrointestinal Malignancies

FDG PET/CT is reported to have variable sensitivity for the detection of primary gastrointestinal (GI) tumors and their nodal, liver, and peritoneal metastases. The low tumor cell densities and metabolic and proliferative rates of some histological subtypes (such as mucinous and signet-ring cell tumors) are responsible for false-negative findings on FDG PET/CT. Multiple conditions such as diverticulitis, inflammatory bowel disease, tuberculosis, post-surgical inflammatory changes, anastomotic site/fistula-related and physiological bowel uptake lower the specificity of FDG PET/CT for GI malignancies [71].

**Figure 3:**
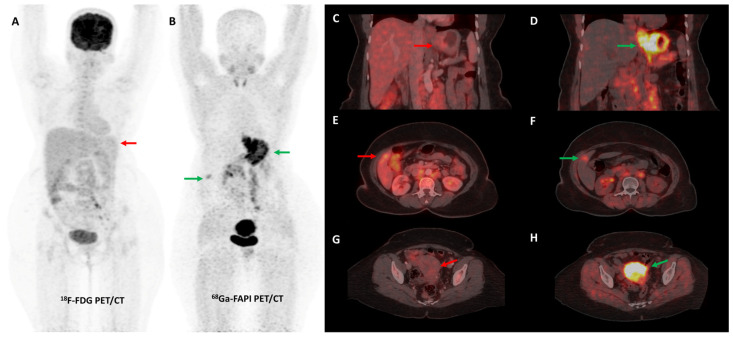
FDG and [^68^Ga]Ga-FAPI-04 PET/CT images in a 42-year-old woman with biopsy-proven gastric adenocarcinoma. The primary lesion in the stomach showed no abnormal FDG uptake ((**A**,**C**)—red arrows) with intense [^68^Ga]Ga-FAPI-04 tracer avidity ((**B**,**D**)—green arrows). FAPI PET/CT revealed a tracer avid hypodense lesion in segment V of the liver ((**B**,**F**)—green arrows), which was not picked up on FDG PET/CT ((**E**)—red arrow), leading to upstaging of disease. Additionally, the uterus showed no abnormal FDG uptake ((**G**)—red arrow) but had diffuse intense FAPI uptake ((**H**)—green arrow), which was interpreted as physiologic/benign uptake.

**Figure 4:**
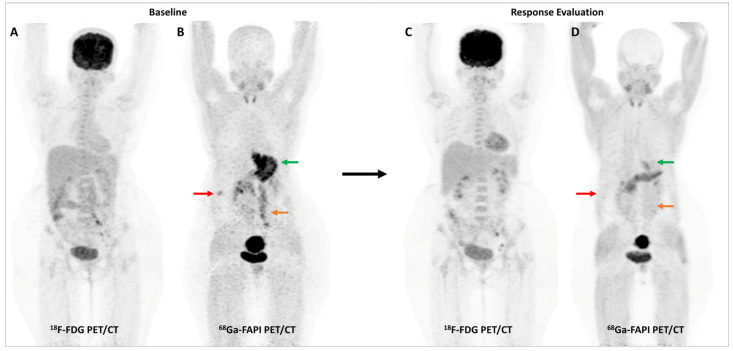
Incremental role of [^68^Ga]Ga-FAPI-04 PET/CT over FDG PET/CT in a 42-year-old woman with metastatic gastric adenocarcinoma for post-chemotherapy response assessment. Baseline (**A**) and follow-up (**C**) FDG PET/CT scans did not reveal significant abnormal tracer uptake in the primary and metastatic lesions. Baseline [^68^Ga]Ga-FAPI-04 PET/CT (**B**) showed tracer avid gastric primary (green arrow), abdominal lymph nodes (orange arrow), and solitary liver metastasis (red arrow). Post-chemotherapy [^68^Ga]Ga-FAPI-04 PET/CT (**D**) demonstrated minimal tracer avidity in the gastric primary (green arrow) with resolution of tracer avidity in the abdominal lymph nodes (orange arrow) and liver lesion (red arrow), suggesting a favorable response to treatment.

Activation of CAFs, particularly in the early development phase, leads to FAP overexpression both in tumor cells and the surrounding stromal component in CRC and other GI malignancies [72]. A higher degree of FAP expression is associated with higher tumor grade, invasiveness, and poor prognosis in CRC [73]. The first real-world clinical experience with FAP-targeted PET imaging (using either [^68^Ga]Ga-FAPI-04 or [^68^Ga]Ga-FAPI-46) in lower GI tract malignancies was reported by a German group. They found significant tracer uptake in anal cancer and liver metastases, with the TBR of most lesions being greater than 3. TNM staging was altered in 50% of the treatment-naïve patients and additional findings were picked up in 47% of patients with metastases. FAPI PET/CT improved TVD in a majority of patients being planned for radiotherapy (RT) [74]. Kömek et al. performed a head-to-head comparison of [^68^Ga]Ga-FAPI-04 and FDG PET/CT in 39 CRC patients. They found that FAPI PET/CT had higher sensitivity and specificity in the detection of primary lesions and lymph nodal metastases [75]. Pang et al. compared the diagnostic performance of [^68^Ga]Ga-FAPI-04 and FDG PET/CT in 35 patients with CRC, gastric and duodenal cancers. Overall, FAPI PET/CT had better sensitivity than FDG PET/CT for the detection of primary tumors (100% vs. 53%, respectively, *p* = 0.004), nodal (79% vs. 54%, *p* = 0.001), and distant metastases (89% vs. 57%, *p* = 0.001). FAPI PET/CT led to upstaging of the clinical TNM stage in 21% of the treatment-naïve patients [76]. Lin et al. reported the additional utility of [^68^Ga]Ga-FAPI-04 PET/CT in monitoring response to treatment in gastric carcinoma [77]. One such demonstrative case of gastric cancer is presented in Figure 3 and Figure 4.

**Figure 5:**
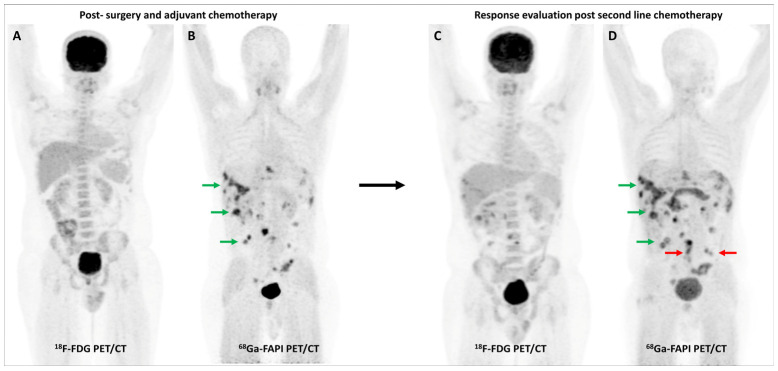
FDG and [^68^Ga]Ga-FAPI-04 PET/CT in a 33-year-old man with histopathologically proven mucinous adenocarcinoma of appendix post cytoreductive surgery, hyperthermic intraperitoneal chemotherapy, and platin-based adjuvant chemotherapy. FDG PET/CT (**A**) did not reveal significant abnormal tracer uptake. However, FAPI PET/CT (**B**) performed two days later revealed multiple tracer avid paracolic gutter, omental, peritoneal, and liver metastases (green arrows). Palliative chemotherapy with capecitabine and oxaliplatin was started. Subsequent response assessment was performed after 3 cycles of this second-line chemotherapy. FDG PET/CT (**C**) underestimated disease burden when compared with FAPI PET/CT (**D**), which showed few new peritoneal deposits (red arrows) in addition to pre-existing lesions (green arrows), suggestive of disease progression. This impacted management as the patient was started on third-line agents, irinotecan and panitumumab.

Multiple studies have consistently reported that FAP-targeted PET/CT imaging improves the detection of malignant peritoneal involvement, which is often challenging to pick up on conventional imaging [78,79]. A meta-analysis (including 11 studies and 340 patients) reported that [^68^Ga]Ga-FAPI PET/CT had significantly higher pooled sensitivity than FDG PET/CT for the detection of peritoneal metastases on both patient-based (98.2% vs. 55.9%) and lesion-based (99.9% vs. 27.3%) analyses [80]. Figure 5 highlights a representative case with peritoneal involvement.

Zhao et al. evaluated the role of [^68^Ga]Ga-FAPI-04 PET/CT for RT planning in 21 patients with locally advanced esophageal cancer. Tracer uptake in the primary tumors was significantly higher on FAPI PET than on FDG PET (median SUVmax 16.7 vs. 11.2, *p* = 0.002). FAPI-based RT planning allows for more accurate TVD, helps avoid tumor geographic misses, and may potentially impact treatment outcomes [81]. In light of preliminary evidence, the adoption of FAPI PET/CT into routine clinical practice for imaging GI malignancies appears imminent.

### 5.4. Brain Tumors

High physiologic uptake is a major limitation to the utility of FDG PET/CT in brain tumors. FAPI PET/CT can be a game-changer in this regard, owing to its low physiologic background activity in the brain. FAP expression levels correlate with tumor grade in glial tumors. Benign brain lesions and low-grade astrocytomas show negligible FAP expression [82,83,84].

Röhrich et al. studied the pharmacokinetics of [^68^Ga]Ga-FAPI-02 and [^68^Ga]Ga-FAPI-04 in mice transfected with human glioblastoma cell line U87MG. Both radiotracers showed robust accumulation in the tumor xenografts within 20 min. Clinical PET imaging was performed in 18 glioma patients, 13 of which were isocitrate dehydrogenase (IDH)-wildtype and 5 were IDH-mutant. IDH-wildtype glioblastomas and grade III/IV IDH-mutant gliomas demonstrated significant tracer uptake with high TBRs, enabling distinction between low-grade and high-grade gliomas. However, it remained unclear whether the heterogeneous uptake pattern in individual tumors was a result of local differences in tumor perfusion or due to intratumoral inhomogeneity of FAP expression [85]. In a subsequent study, the same group correlated FAP-specific signaling with apparent diffusion coefficient (ADC) and relative cerebral blood volume (rCBV) signals on MRI to further characterize the significance of FAPI tracer uptake ([^68^Ga]Ga-FAPI-02 or [^68^Ga]Ga-FAPI-04) in 13 patients with IDH-wildtype glioblastomas. FAP-specific PET signaling in glioblastomas showed only a moderately positive correlation with rCBV and no correlation with ADC, indicating that FAPI PET imaging is primarily not a surrogate marker of perfusion or cell density, and provides complementary information to MRI [86]. Yao et al. evaluated the role of [^18^F]F-NOTA-FAPI-04 PET/CT in 12 glioblastoma patients before RT. [^18^F]F-FAPI PET/CT revealed tracer avidity in 16 out of 23 lesions described on MRI (mean SUVmax 7.08 and mean TBR 19.95). Ki-67 index and molecular expression profile did not show any significant correlation with FAPI PET/CT parameters. Despite a moderate sensitivity (69.6%), the excellent positive predictive value (PPV) (100%) of FAPI PET/CT may help when MRI findings are equivocal and can potentially guide biopsy site selection [87]. High TBRs allow the application of FAPI PET/CT (using either [^68^Ga]Ga-FAPI-02 or [^68^Ga]Ga-FAPI-04) for RT planning in glioblastoma patients as demonstrated by Windisch et al. in a pilot study. FAPI PET-based TVD caused no complications and resulted in GTVs containing tumors not covered by MRI-GTVs [88].

### 5.5. Head and Neck Cancer

The use of FDG PET/CT in head and neck cancers (HNCs) is limited by its low specificity. Multiple sources of false positives include brown fat uptake, non-specific/exertional FDG uptake in cervical muscles, variable salivary gland uptake, post-surgical changes, RT sequelae, and other inflammatory pathologies. Accurate assessment of disease extent, particularly infiltration of complex peritumoral structures, remains challenging [89]. FAPI PET/CT may overcome some of these limitations due to its higher image contrast.

In an early study by Syed et al., 14 patients with HNCs demonstrated high [^68^Ga]Ga-FAPI tracer avidity in the primary tumors (mean SUVmax 14.6 ± 4.4) with low background uptake. Automated FAPI PET/CT-based GTVs were significantly different from GTVs manually contoured on CECT images by experienced radiation oncologists [90]. Serfling et al. reported that [^68^Ga]Ga-FAPI-04 PET/CT improved primary tumor localization in patients with carcinoma of unknown primary or suspected Waldeyer’s ring malignancy, having higher TBRs than FDG (mean TBR 10.9 vs. 4.1). However, FAPI PET/CT had a lower detection rate of cervical nodal metastases than FDG PET/CT [91]. A head-to-head comparison of [^68^Ga]Ga-FAPI-04 and FDG PET/MR in patients with nasopharyngeal carcinoma demonstrated the superiority of FAPI over FDG in detecting skull base and intracranial invasion. FAPI changed overall staging in 40% (6/15) of patients compared with FDG (3—upstaged and 3—downstaged). But a limitation of FAPI PET was its lower sensitivity in identifying nodal metastases compared to FDG [92]. Despite its lower sensitivity, [^68^Ga]Ga-FAPI PET/CT was found to have significantly higher specificity and overall accuracy than FDG PET/CT for preoperative nodal staging (patient-based analysis) in 36 patients with oral squamous cell carcinoma [93]. Significant FAPI tracer ([^68^Ga]Ga-FAPI-02, [^68^Ga]Ga-FAPI-46 or [^68^Ga]Ga-FAPI-74) accumulation (mean SUVmax 11.16 ± 4.07 and mean TBR 6.64 ± 3.13) was reported in primary adenoid cystic carcinoma (ACC) by Röhrich et al. In 41.7% (5/12) of patients, FAPI PET/CT upstaged the disease compared to conventional imaging. FAPI PET/CT-based GTVs were found to be more accurate than those manually contoured based on CECT or CE-MRI images, allowing precise RT plans, including areas of intra-cranial extension and perineural tumor invasion [94].

### 5.6. Thyroid Cancer

FDG PET/CT is particularly important for recurrence evaluation in differentiated thyroid cancer (DTC) patients having thyroglobulin elevation with negative iodine scintigraphy (TENIS) [95]. FDG PET/CT is also widely used to assess disease extent in patients with radioiodine-refractory DTC (RR-DTC) [96]. Given limited systemic therapeutic options, multiple tracers, including radiolabeled FAPIs have been tried in progressive RR-DTC with the ultimate aim of potential theranostics [97].

The presence of CAFs in tumor stroma positively correlates with dedifferentiation and aggressive outcomes of thyroid cancer [98]. Fu et al. reported the first case of DTC with TENIS demonstrating tracer avid local recurrence and distant metastases on [^68^Ga]Ga-FAPI-04 PET/CT [99]. Subsequently, the same group reported additional metastatic lesions seen with [^68^Ga]Ga-FAPI-04 but not with FDG PET/CT in a patient with RR-DTC, owing to its better lesion-to-background ratio [100]. Prompted by these encouraging findings, Fu et al. prospectively assessed the clinical utility of [^68^Ga]Ga-FAPI-04 PET/CT in 35 metastatic DTC patients. Overall, tracer avidity in most metastatic DTC lesions was higher on FAPI PET/CT than FDG PET/CT, being significantly more for lateral compartment cervical, axillary and mediastinal lymph nodes, and pulmonary metastases. FAPI PET/CT was found to have higher sensitivity than FDG PET/CT for detecting cervical lesions (83% vs. 65%, *p* = 0.01) and distant metastases (79% vs. 59%; *p* < 0.001), on a lesion-based analysis [101]. Mu et al. evaluated the utility of [^18^F]F-FAPI-42 PET/CT in DTC patients with TENIS and found its performance comparable to FDG PET/CT [102]. Few case reports have also explored the role of FAP-targeted PET imaging and theranostics in medullary and anaplastic thyroid cancer [103,104].

### 5.7. Breast Cancer and Other Gynecological Malignancies

**Figure 6:**
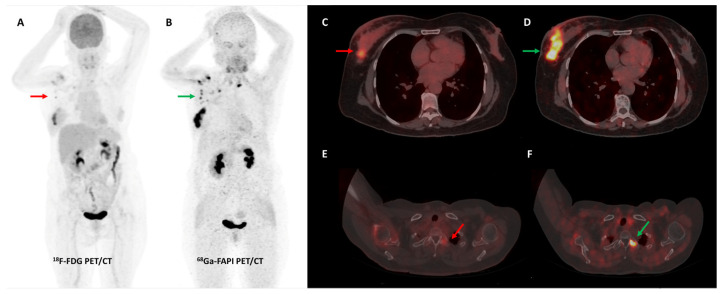
[^68^Ga]Ga-FAPI-04 performed better than FDG PET/CT for initial staging in a 51-year-old woman with histopathologically proven triple receptor-negative invasive breast cancer. FDG PET/CT (**A**) revealed mildly tracer avid right breast primary ((**C**)—red arrow) with few faintly FDG avid right axillary lymph nodes ((**A**)—red arrow). FAPI PET/CT (**B**) demonstrated intensely tracer avid right breast primary ((**D**)—green arrow), multiple right axillary lymph nodes ((**B**)—green arrows), and few lytic skeletal lesions such as one involving the left transverse process of C2 vertebra ((**F**)—green arrow), which showed no abnormal FDG uptake ((**E**)—red arrow).

Breast cancer is characterized by a remarkable degree of genetic and molecular heterogeneity. Differences in receptor status expression are fundamental to the biological behavior of breast cancer subtypes, directly impacting imaging and treatment strategies [105]. In recent years, radiotracers specific to the estrogen receptor (ER) and human epidermal growth factor receptor 2 (HER2) such as [^18^F]fluoroestradiol (FES) and [^89^Zr]Zr-Trastuzumab, respectively, have demonstrated clinical utility [106,107]. FAP targeting represents a promising approach to indirectly assess tumor burden as CAFs are the most abundant cells in the breast cancer microenvironment. Komek et al. conducted a pilot study, prospectively comparing [^68^Ga]Ga-FAPI-04 and FDG PET/CT in 20 women with histopathologically confirmed breast cancer. FAPI had higher sensitivity (100% vs. 78.2%) than FDG PET/CT, with comparable specificity (96.5% vs. 100%) for the detection of primary breast lesions. The SUVmax values of primary breast lesions, lymph nodal, lung, and bone metastases were significantly higher with FAPI than FDG (*p* < 0.05) [108]. Figure 6 shows a representative example.

**Figure 7:**
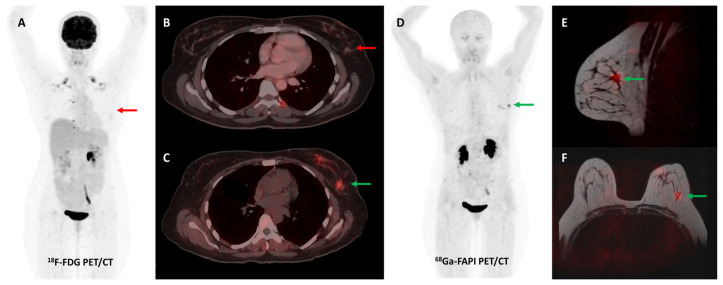
Forty-four-year-old woman, a known case of epithelial ovarian carcinoma post hysterectomy and bilateral salpingo-oophorectomy, underwent FDG and [^68^Ga]Ga-FAPI-04 PET/CT for restaging. FAPI tracer avid ((**C**,**D**)—green arrows) and non-FDG avid ((**A**,**B**)—red arrows) ill-defined soft tissue density nodule (~1 × 0.8 cm) was noted in the upper outer quadrant of the left breast, which was corroborated on the fused PET-MR mammogram images ((**E**,**F**)—green arrows). Subsequently, an ultrasound-guided left breast biopsy was performed which showed invasive lobular carcinoma with ER/PR positive and HER2 negative receptor status.

A retrospective study by Elboga et al. found that [^68^Ga]Ga-FAPI-04 PET/CT picked up more lesions and showed higher tracer avidity than FDG PET/CT in 48 invasive breast cancer patients. In the post-chemotherapy group, 12/24 (50%) patients who were classified as having disease control on FDG PET/CT were re-classified as having progressive disease according to FAPI PET/CT-based response evaluation [109]. A German group retrospectively evaluated the role of [^68^Ga]Ga-FAPI-46 breast PET/MRI and whole-body PET/CT in 19 women with biopsy-proven invasive breast cancer. A high degree of tracer accumulation was noted in all primary tumor lesions (mean SUVmax 13.9 ± 5.6) and pre-operatively verified axillary lymph nodes (mean SUVmax 12.2 ± 6.2). No significant difference in tracer uptake of primary tumors was noted across different grades, receptor statuses, or histologic types [110]. A prospective pilot trial evaluated the role of [^68^Ga]Ga-FAPI-04 PET/CT in a small sample of 7 patients with non-FDG-avid metastatic invasive lobular breast cancer. FAPI PET/CT had significantly higher lesion detection when compared with CT alone (*p* = 0.022). FAPI PET/CT was particularly useful to detect infiltrative soft tissue and serosal involvement [111]. Figure 7 shows an interesting example of the utility of FAPI PET/CT in a patient with invasive lobular breast cancer.

Dendl et al. reported impressive lesional SUV metrics with ^68^Ga-labeled FAP ligands (FAPI-02, FAPI-04, FAPI-46, or FAPI-74) in a heterogeneous cohort of 31 patients having various gynecological malignancies. TBRs for distant metastases were significantly higher on FAPI than FDG PET/CT (13.0 vs. 5.7, *p* = 0.047). IHC staining demonstrated strong FAP expression in ovarian cancer, breast cancer, and uterine leiomyosarcoma. The authors also reported that physiologic/benign FAPI uptake in the endometrium and breast was significantly different between premenopausal and post-menopausal women of a different cohort, having various tumor entities [112].

**Figure 8:**
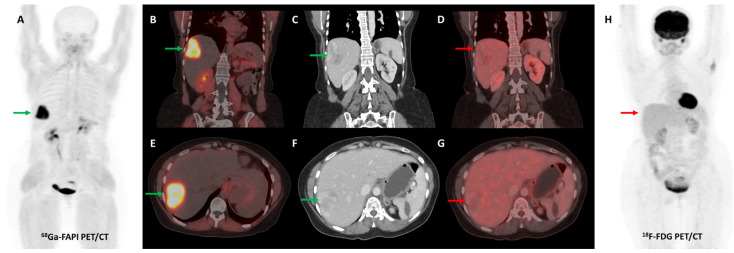
Forty-seven-year-old woman, a known case of ovarian carcinoma (clear cell type) post-surgery and adjuvant platin-based chemotherapy had rising serum CA-125 levels. She underwent [^68^Ga]Ga-FAPI-04 and FDG PET/CT for restaging, which revealed a heterogeneously enhancing lesion (~5.1 × 3.5 cm) in segment VII of the liver ((**C**,**F**)—green arrows) with intense FAPI uptake ((**A**,**B**,**E**)—green arrows) and no significant FDG uptake ((**D**,**G**,**H**)—red arrows) suggestive of liver metastasis.

Several preclinical studies have demonstrated FAP expression in a vast majority (>90%) of ovarian cancers with negligible expression in normal ovarian tissue, benign and borderline tumors. FAP expression in epithelial ovarian cancers is associated with resistance to platinum-based chemotherapy, shorter time to recurrence, and overall worse clinical outcomes [113,114,115,116]. In a recent study, [^68^Ga]Ga-FAPI-04 showed negligible physiological accumulation in ovaries irrespective of the menstrual cycle phase, suggesting that it could overcome limitations associated with non-specific/benign ovarian FDG uptake [117]. Zheng et al. retrospectively compared [^68^Ga]Ga-FAPI-04 and FDG PET/CT in 21 patients with suspected (*n* = 11) or already diagnosed (*n* = 10) primary ovarian malignancy. FAPI was more sensitive than FDG PET/CT for the detection of primary tumors (100% vs. 78%), nodal metastases (100% vs. 80%), and peritoneal/pleural involvement (100% vs. 56%), leading to upstaging of disease in 19% (4/21) of the patients [118]. Figure 8 shows an example of FAPI PET/CT in a patient with ovarian malignancy.

### 5.8. Sarcoma

Both neoplastic and stromal cells show FAP expression in sarcomas [13]. An early study by Koerber et al. reported high lesional SUVmax values and excellent TBRs (>7) with different ^68^Ga-labeled FAP ligands in patients with different sarcomas. Higher uptake was noted with high-grade tumors and clinically more aggressive disease [119]. Kessler et al. further explored the role of [^68^Ga]Ga-FAPI-46 PET/CT in 46 patients with bone and soft-tissue sarcomas, as a subgroup analysis of an ongoing prospective observational trial (NCT04571086). FAPI PET-SUVmax and histopathological FAP expression showed a significant, moderate linear association (Spearman r = 0.43, *p* < 0.05). In FAPI PET-positive patients with histopathologic validation, the PPV was 1.00 on a per-patient basis and 0.97 on a per-region basis. Detection rates of FAPI PET/CT and FDG PET/CT were comparable on a per-patient basis (97.7% vs. 95.3%, respectively). FAPI PET upstaged the disease in 18.6% of patients when compared with FDG PET. FAPI PET/CT changed clinical management in 30% of patients [120]. Gu et al. demonstrated the superiority of [^68^Ga]Ga-FAPI-04 PET/CT over FDG PET/CT in patients with soft-tissue sarcoma. Overall, FAPI detected more lesions than FDG PET/CT (275 vs. 186) and had significantly higher sensitivity, specificity, and accuracy for the diagnosis of recurrent lesions (All *p* values < 0.001) [121].

### 5.9. Pitfalls

Physiological FAP expression in adult healthy tissues is negligible [16]. However, the demonstration of FAP expression on activated fibroblasts involved in wound healing, tissue remodeling, fibrosis, degenerative, arthritic processes, and atherosclerosis, has raised concerns over the specificity of FAPI PET as an imaging modality for malignancies. A recent study by Kessler et al. reported that degenerative lesions in joints and vertebral bones were the most common cause of non-tumor-specific tracer uptake on [^68^Ga]Ga-FAPI (FAPI-04 or FAPI-46) PET/CT, being seen in more than 50% of patients. Variable physiological uterine tracer uptake has also been reported in a majority of women, being significantly higher in the premenopausal setting, hindering the interpretation of FAPI PET/CT in gynecological malignancies. Other reported sites of non-tumor-specific uptake include muscles, scarring/wounds, oral/nasal mucosa, salivary glands, teeth, and mammary glands [122]. Several case reports have highlighted FAPI uptake in benign tumors such as cutaneous fibroma, schwannoma, renal angiomyolipoma, pulmonary solitary fibrous tumor, and others [123,124,125,126]. FAPI tracer uptake has also been reported in inflammatory conditions such as myocarditis, pneumonitis, pleuritis, appendicitis, colitis, and sclerosing cholangitis, to name a few [127,128,129,130]. Additionally, post-chemotherapy, RT, and surgery-induced inflammation and fibrotic changes can show FAPI tracer uptake [131]. Accurate interpretation of FAPI PET/CT studies is heavily reliant on the familiarity of reporting physicians with the above-mentioned pitfalls.

## 6. FAPI PET/CT—Non-Oncological Indications

As previously mentioned, FAP overexpression is not specific to CAFs. There is an emerging body of evidence that the appropriate use of FAPI PET/CT may find application in a wide range of non-oncological pathological states.

### 6.1. Cardiovascular Diseases

A retrospective study by Heckmann et al. first reported an association between left ventricular ^68^Ga-labeled FAPI radiotracer uptake and cardiovascular risk factors such as obesity, diabetes mellitus, and radiation exposure to the chest [132]. Another retrospective study with a limited sample size found that higher myocardial ^68^Ga-labeled FAPI radiotracer uptake was associated with clinical and serum markers of myocarditis in patients undergoing immune-checkpoint inhibitor therapy [127]. Wang et al. demonstrated intense and inhomogeneous myocardial FAPI tracer uptake in patients with hypertrophic cardiomyopathy (HCM), which was higher than that of healthy controls. Additionally, a positive correlation was observed between the degree of ^18^F-labeled FAPI cardiac activity and the 5-year risk of sudden cardiac death (SCD) (*r* = 0.32, *p* = 0.03) [133]. Siebermair et al. reported a case of a 58-year-old lady with cardiac sarcoidosis in whom fibroblast activity demonstrated by [^68^Ga]Ga-FAPI-46 PET/CT suggested ongoing cardiac remodeling and guided continuation of immunomodulatory therapy with steroids and azathioprine [134]. Tillmanns et al. first reported increased levels of FAP expression in murine and human hearts post-myocardial infarction (MI), typically in the peri-infarct zone and reaching peak levels approximately seven days post-MI [135]. Diekmann et al. sought to understand the significance of early cardiac fibroblast activation in acute MI and its correlation with subsequent functional outcomes. Their retrospective study included 35 patients who underwent resting myocardial perfusion SPECT, [^68^Ga]Ga-FAPI-46 PET/CT, and cardiac magnetic resonance (CMR) imaging within 11 days after reperfusion therapy for acute MI. The PET-based extent of FAP upregulation was significantly larger than SPECT perfusion defect size (58 ± 15 vs. 23 ± 17%, *p* < 0.001) and CMR-based infarct area (28 ± 11%, *p* < 0.001). Only a weak correlation was observed between cardiac FAP-volume and baseline left ventricular ejection fraction (LVEF) (r = −0.32, *p* = 0.07). However, cardiac FAP-volume had a significant inverse correlation with LVEF obtained at later follow-up (r = −0.58, *p* = 0.007) [136]. Similar findings were also reported by Xie et al. [137]. These results suggest that FAPI PET/CT may provide novel imaging biomarkers predictive of ventricular remodeling post-acute MI. A single-center pilot study also reported the utility of ^68^Ga-labeled FAPI PET/CT in non-invasive visualization of fibrotic remodeling of the right ventricle in patients with pulmonary arterial hypertension [138].

### 6.2. Liver Fibrosis and Cirrhosis

FAP expression has been reported on activated hepatic stellate cells and is believed to play a role in tissue remodeling and ECM changes in liver fibrosis and cirrhosis [139]. Pirasteh et al. reported a strong correlation between [^68^Ga]Ga-FAPI-46 uptake in the liver and the histologic stage of liver fibrosis in a preclinical swine model [140]. Few clinical studies have reported elevated hepatic parenchymal [^68^Ga]Ga-FAPI-04 tracer uptake in patients with cirrhosis compared to those without cirrhosis, suggesting that co-existing cirrhosis may potentially hinder the interpretation of FAPI PET/CT performed for evaluation of primary liver malignancies [61].

### 6.3. Arthritic Disorders

Preliminary case reports have demonstrated FAPI tracer uptake in degenerative and inflammatory arthritic disorders, which can possibly be explained by higher levels of FAP expression on fibroblast-like synoviocytes (FLSs) and chondrocytes [141,142,143,144]. A pre-clinical study revealed higher in- vitro binding of [^18^F]AlF-NOTA-FAPI-04 to FLSs of rheumatoid arthritis (RA) compared to controls [145]. In a recent prospective study, Luo et al. evaluated the role of ^68^Ga-labeled FAPI PET/CT in twenty patients with RA. FAPI PET/CT demonstrated 244 affected joints, 6.1% of which were negative on FDG PET/CT. SUVmax of the most affected joint was significantly higher on FAPI than FDG PET/CT (9.54 ± 4.92 vs. 5.85 ± 2.81, respectively, *p* = 0.001). Additionally, the PET joint count and PET articular index scores with FAPI showed a significant positive correlation with most clinical disease activity variables and radiographic progression of joint damage (*p* < 0.05) [146].

### 6.4. IgG4-Related Disease

IgG4-related disease (IgG4-RD) is an immune-mediated multi-system disorder typically characterized by IgG4-positive plasma cell-rich lymphoplasmacytic infiltration, varying degrees of storiform fibrosis, and serum IgG4 elevation [147]. Encouraged by early case reports, Luo et al. prospectively evaluated the role of [^68^Ga]Ga-FAPI-04 PET/CT in 26 patients with IgG4-RD and found that it had overall higher lesion detection rates compared to FDG PET/CT, particularly for the detection of pancreatic, lacrimal gland, and hepatobiliary involvement. However, FDG was superior for the detection of lymph nodal involvement [148,149]. Another study from Germany found that a combination of [^68^Ga]Ga-FAPI-04 and FDG PET/CT might assist in distinguishing fibrotic and inflammatory activity in IgG4-RD. This could impact the approach to treatment, such as choosing anti-fibrotic drugs instead of broad-spectrum anti-inflammatory medications in patients who have predominant fibrosis with negligible/low-grade active inflammation [150].

### 6.5. Pulmonary Fibrosis and Interstitial Lung Disease

Rosenkrans et al. demonstrated that [^68^Ga]Ga-FAPI-46 PET imaging was superior to CT for the identification and monitoring of lung fibrogenesis in a preclinical murine model of bleomycin-induced pulmonary fibrosis [151]. A pilot study by Bergmann et al. demonstrated the utility of [^68^Ga]Ga-FAPI-04 PET/CT in 21 patients with systemic sclerosis-associated interstitial lung disease (ILD). FAPI tracer uptake was higher in patients with extensive/progressive/active disease compared to those with limited/stable/inactive disease, respectively. Additionally, change in FAPI tracer uptake was concordant with the therapeutic response to the fibroblast-targeting antifibrotic drug, nintedanib. Increased FAPI uptake at baseline was associated with ILD progression independent of baseline CT-disease extent and forced vital capacity [152]. In an exploratory study by Röhrich et al. [^68^Ga]Ga-FAPI-46 PET/CT demonstrated markedly elevated tracer uptake and high TBRs in 15 patients with fibrosing ILDs (fILDs) and suspected lung cancer. A positive association was observed between FAPI tracer uptake and CT-based fibrosis index. Moreover, time activity curves obtained from dynamic FAPI PET imaging permitted differentiation between fILD and lung cancer [153].

### 6.6. Others

Luo et al. demonstrated intense FAPI tracer uptake by the involved bowel in Crohn’s disease (CD) but not in ulcerative colitis [154]. FAP expression is notably increased in the intestinal myofibroblasts located in the muscle layer of CD strictures [155]. Chen et al. conducted a pilot study evaluating the role of [^68^Ga]Ga-FAPI-04 PET/CT in 16 patients with CD. FAPI PET/CT was superior to CT enterography (CTE) in the detection of endoscopic lesions (*p* < 0.05). FAPI PET/CT correlated well with endoscopic, CTE, clinical, and biochemical markers of CD [156]. A few case reports have demonstrated the utility of FAPI PET/CT in the evaluation of chronic granulomatous inflammatory disorders such as tuberculosis and sarcoidosis [134,157,158]. Few studies have assessed the role of ^68^Ga-labeled FAPI PET/CT in evaluating periprosthetic joint infections and aseptic loosening and revealed that FAPI could define the extent of such lesions more accurately than FDG [159,160]. Overall, the application of FAPI PET/CT in benign inflammatory, infectious, and immune pathologies is in its nascent stages and requires large-scale prospective evaluation before meaningful translation to clinical practice.

## 7. Conclusions and Future Directions

Based on this review of existing literature, preliminary data on FAPI PET/CT remains encouraging, demonstrating utility for a diverse range of oncological and non-oncological indications. Nuclear medicine physicians across the globe must shoulder the responsibility to conduct well-structured and focused clinical trials that will help us identify appropriate indications and clinical scenarios for the use of FAP-targeted imaging. The goal is not to replace the current standard FDG PET/CT but to understand how the complementary information provided by FAPI PET/CT can be utilized to potentially impact clinical decision-making and management protocols. Additionally, the realm of FAP-targeted nuclear theranostics is intriguing and warrants further exploration as it can open up a new avenue for patients who have progressed despite conventional treatment modalities.

## Data Availability

Not applicable.
